# Awakening the endogenous Leloir pathway for efficient galactose utilization by *Yarrowia lipolytica*

**DOI:** 10.1186/s13068-015-0370-4

**Published:** 2015-11-25

**Authors:** Zbigniew Lazar, Heber Gamboa-Meléndez, Anne-Marie Crutz- Le Coq, Cécile Neuvéglise, Jean-Marc Nicaud

**Affiliations:** INRA, UMR1319 Micalis, 78352 Jouy-en-Josas, France; AgroParisTech, UMR Micalis, 78352 Jouy-en-Josas, France; Department of Biotechnology and Food Microbiology, Wroclaw University of Environmental and Life Sciences, Chełmońskiego 37/41, 51-630 Wroclaw, Poland

**Keywords:** Galactose, GAL genes, Citric acid, Lipid accumulation, Single-cell oil, Yeasts, Metabolic pathways

## Abstract

**Background:**

Production of valuable metabolites by *Yarrowia lipolytica* using renewable raw materials is of major interest for sustainable food and energy. Galactose is a monosaccharide found in galactomannans, hemicelluloses, gums, and pectins.

**Results:**

*Yarrowia lipolytica* was found to express all the Leloir pathway genes for galactose utilization, which encode fully functional proteins. Gene organization and regulation in *Y. lipolytica* resembles filamentous fungi rather than *Saccharomyces cerevisiae*. After *Y. lipolytica* was grown on mixture of glucose and galactose, it was then able to metabolize galactose, including when glucose concentrations were higher than 4 g/L. However, glucose was still the preferred carbon source. Nonetheless, a strain overexpressing the four *ylGAL* genes of the Leloir pathway was able to efficiently use galactose as its sole carbon source. This mutant was used to produce citric acid and lipids from galactose; the yields were comparable to or greater than that obtained for the parental strain (W29) on glucose.

**Conclusions:**

The construction of a *Y. lipolytica* strain able to produce citric acid and lipids from galactose is a very important step in bypassing issues related to the use of food-based substrates in industrial applications.

**Electronic supplementary material:**

The online version of this article (doi:10.1186/s13068-015-0370-4) contains supplementary material, which is available to authorized users.

## Background

**Fig. 1:**
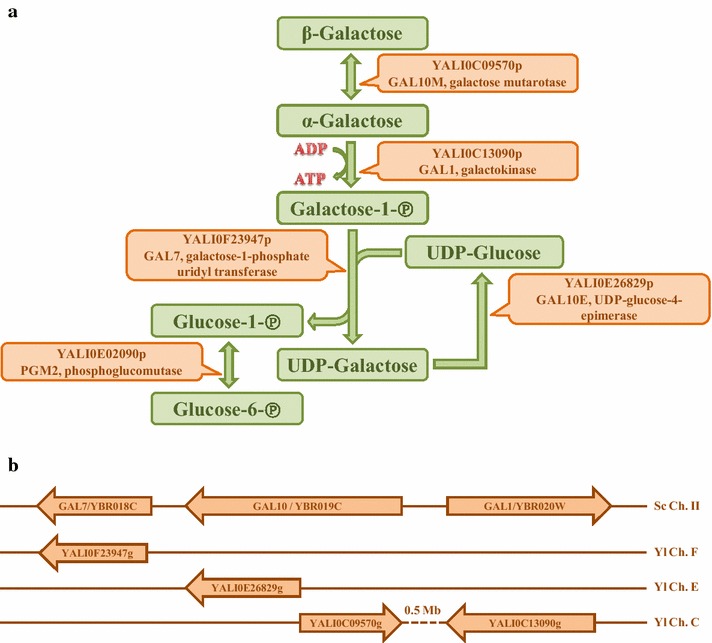
Schematic representation of the Leloir pathway of galactose metabolism in *Y. lipolytica* (**a**), and GAL genes organization in the *S. cerevisiae* and *Y. lipolytica* genomes (**b**)

Compounds such as citric acid, lipids, or sugar alcohols are of industrial interest; as a consequence, using microbes such as *Yarrowia lipolytica* to produce them has spurred research around the world [1–3]. From an economic and social point of view, it is very important to identify raw materials that could serve as cheap and renewable substrates and that are not already in demand for food production. Therefore, at present, research efforts are focused on using plant biomass, which contains a diversity of sugars, for the production of these valuable compounds [4]. One such sugar is d-galactose, a monosaccharide that is a C4 epimer of glucose. The polysaccharides found in plant cell walls (e.g., galactomannans), gums, hemicelluloses, and pectins are rich sources of galactose [5]. Galactose also occurs naturally in milk; lactose is made up of galactose and glucose. Both milk and plant biomass are readily exploited by many microorganisms, including bacteria, yeasts, and fungi. These diverse species utilize similar pathways to break down galactose. For example, the bacterium *Azotobacter vinelandii* uses the non-phosphorylative DeLey-Doudoroff pathway to metabolize galactose. Galactose is oxidized, forming galactonate, which is ultimately broken down into pyruvate and glyceraldehyde 3-phosphate [6]. Another pathway, the oxido-reductive pathway, exists in filamentous fungi such as *Aspergillus niger* or *Hypocrea jecorina*; it involves a series of redox reactions that convert galactose to d-fructose [7]. However, the best-known and best-studied pathway is the Leloir pathway, which occurs in many organisms, including yeasts (e.g., *Saccharomyces cerevisiae* and *Kluyveromyces lactis*) [8, 9]. Via this phosphorylative pathway, d-galactose is converted to glucose-6-phosphate (Fig. 1a). In *S. cerevisiae*, β-d-galactose is first converted to its α-anomer by galactose mutarotase (*scGAL10*, YBR019C); only this anomeric form can be utilized by cells. Subsequently, α-d-galactose is phosphorylated by galactokinase (*scGAL1*, YBR020W), releasing galactose-1-phosphate. Then, galactose-1-phosphate uridylyltransferase (*scGAL7*, YBR018C) converts this intermediate compound into UDP-galactose, simultaneously releasing glucose-1-phosphate. In the last step of the pathway, UDP-galactose is epimerized into UDP-glucose by UDP-glucose 4-epimerase (*scGAL10*, YBR019C). It is worth noting that, in *S. cerevisiae* and other Ascomycetes, the epimerase and mutarotase domains are fused together but act independently (Fig. 1b) [10]. In addition, the mutarotase domain is not essential to galactose metabolism because the sugar anomers interconvert spontaneously in water [11]. Phosphoglucomutase converts the glucose-1-phosphate released by the pathway to glucose-6-phosphate, an intermediate compound in glycolysis. However, because phosphoglucomutase is also involved in glycogenolysis, it is not formally considered to be part of the Leloir pathway.

In the well-studied yeast *S. cerevisiae,* the structural *scGAL* genes (*scGAL1, scGAL7, scGAL10*) are clustered and subject to strong transcriptional regulation [9, 12]. Activation of these genes occurs in the presence of galactose, but in the absence of glucose, under the action of regulatory proteins—Gal4p (activator), Gal80p (inhibitor), and Gal3p (activator and ohnolog of *GAL1*, which has a regulatory function but lacks catalytic activity). On the other hand, repression of *scGAL* genes occurs in the presence of glucose and involves, among others, the transcriptional repressor Mig1p [13]. In addition (also in the presence of glucose), galactose uptake is inactivated through degradation of the galactose transporter Gal2p [14]. Unlike *S. cerevisiae*, the Gal3p does not exist in the yeast *K. lactis,* while Gal1p is responsible for both enzymatic and regulatory activities [8]. Furthermore, in the latter yeast, *GAL* genes were shown to be semiconstitutively expressed at low levels and induced during the stationary phase [15].

In filamentous fungi, the Leloir genes are not clustered as they are in *S. cerevisiae* and *K. lacti*s [10]. Moreover, in *H. jecorina*, *A. niger*, and *Aspergillus nidulans*, they exhibited a high basal expression levels on various carbon sources such as glucose, arabinose, xylose, lactose, cellobiose, or glycerol [5, 16–21]. This finding indicates that important differences exist in galactose metabolism regulation in filamentous fungi versus in *S. cerevisiae*. The first and the most significant difference is the presence of at least two different pathways in the genomes of filamentous fungi [7, 17, 18]. Another difference is that proteins that regulate galactose utilization in *S. cerevisiae*—such as Gal4p—are not found in their genomes [16]. However, other proteins regulating galactose metabolism are found in *A. nidulans* (GalR and GalX) [5] and other aspergilli (GalX) [5]. In addition, glucose does not cause carbon catabolite repression of the GAL pathway in filamentous fungi [16, 17]. All these data indicate that the regulation of the Leloir pathway (or its mechanism) described in *S. cerevisiae* is not conserved in other Ascomycetes [16].

*Yarrowia lipolytica* is a dimorphic yeast that belongs to the subphylum Saccharomycotina. Its genome contains all the Leloir genes (Fig. 1a), scattered on different chromosomes as in *H. jecorina* (Fig. 1b) [10]. In addition, the UDP-glucose-4-epimerase and galactose mutarotase are encoded by two different genes in both *Y. lipolytica* and *H. jecorina* [10, 17, 18]. However, the *Y. lipolytica* species is unable to use galactose as its sole carbon source. Therefore, the objective of this study was to figure out whether these genes are even expressed and whether they encode fully functional proteins. The goal was also to determine the culture conditions under which *Y. lipolytica* cells use galactose as a carbon source. Above all, the aim was to construct a *Y. lipolytica* strain that could utilize galactose efficiently and be used for different biotechnological applications. For instance, galactose-containing raw materials, such as polysaccharides obtained from plant cell walls (i.e., hemicelluloses), could eventually supplant food-based substrates in industrial applications.

## Results and discussion

The yeast *Y. lipolytica* is able to use a few monosaccharides as carbon sources, namely glucose, fructose, and mannose [22]. However, in contrast to *S. cerevisiae*, none of the *Y. lipolytica* WT strains are able to utilize pure galactose.

### Structural and regulatory genes for the Leloir pathway in *Yarrowia lipolytica*

The conservation of the Leloir pathway proteins was examined. The proposed nomenclature of the *ylGAL* genes will be used in this paper as follows: *ylGAL1* (YALI0C13090g, galactokinase), *ylGAL7* (YALI0F23947g, galactose-1-phosphate uridyl transferase), *ylGAL10E* (YALI0E26829g, UDP-glucose-4 **E**pimerase), and *ylGAL10M* (YALI0C09570g, galactose **M**utarotase). In *Y. lipolytica*, UDP-glucose-4 epimerase (*ylGAL10E)* is the most conserved of the GAL proteins also found in *S. cerevisiae* and *H. jecorina* (69–76 % amino-acid similarity), whereas galactose mutarotase (*ylGAL10* *M)* is the least conserved (36–42 % similar) (Table 1; http://blast.ncbi.nlm.nih.gov/Blast.cgi). Although the structural genes necessary for galactose utilization are present in *Y. lipolytica*, the genes encoding regulatory proteins in *S. cerevisiae* (i.e., *scGAL3*, *scGAL4*, and *scGAL80*) were not found. Furthermore, *GAL4*-binding sites are not present in the promoter regions of *ylGAL* genes (data not shown), which is also the case for *H. jecorina* [18]. Despite this fact, *H. jecorina* is able to grow well on galactose. Past work has shown that the fission yeast *Schizosaccharomyces pombe* also contains all of the Leloir genes but fails to grow when galactose is the sole available carbon source [23]. The *MIG1* gene (YALI0E07942g), which homolog represses expression of *GAL* genes in *S. cerevisiae*, has been identified in *Y. lipolytica* [24]. Disruption of *ylMIG1* did not result in growth of *Y. lipolytica* on galactose (data not shown).

**Table 1:** Comparison of GAL genes among *Y. lipolytica*, *S. cerevisiae,* and *H. jecorina*

Enzyme name	*Y. lipolytica*			*S. cerevisiae*			Identity (%)	Similarity (%)	*Hypocrea jecorina*			Identity (%)	Similarity (%)
	Abbreviation	Gene	Protein length	Abbreviation	Gene	Protein length			Abbreviation	Locus	Protein length		
Galactokinase	*yl*GAL1	YALI0C13090g	476aa	*sc*GAL1	YBR020w	528aa	40	55	Gal1	XP_006961307	526aa	40	56
Galactose-1-phosphate uridyl transferase	*yl*GAL7	YALI0F23947g	352aa	*sc*GAL7	YBR018c	366aa	49	63	Gal7	XP_006968597	382aa	49	61
UDP-glucose-4-Epimerase	*yl*GAL10E	YALI0E26829g	369aa	*sc*GAL10^a^	YBR019c	699aa	62	76	Gal10E	AF439323_1	370aa	59	69
	*yl*GAL10M	YALI0C09570g	327aa				26	42	Gal10 M	XP_006964994	342aa	29	36

^a^In *S. cerevisiae*, the GAL10 gene contains two domains: epimerase and mutarotase (Fig. 1)

### *Yarrowia lipolytica* GAL genes are expressed

**Fig. 2:**
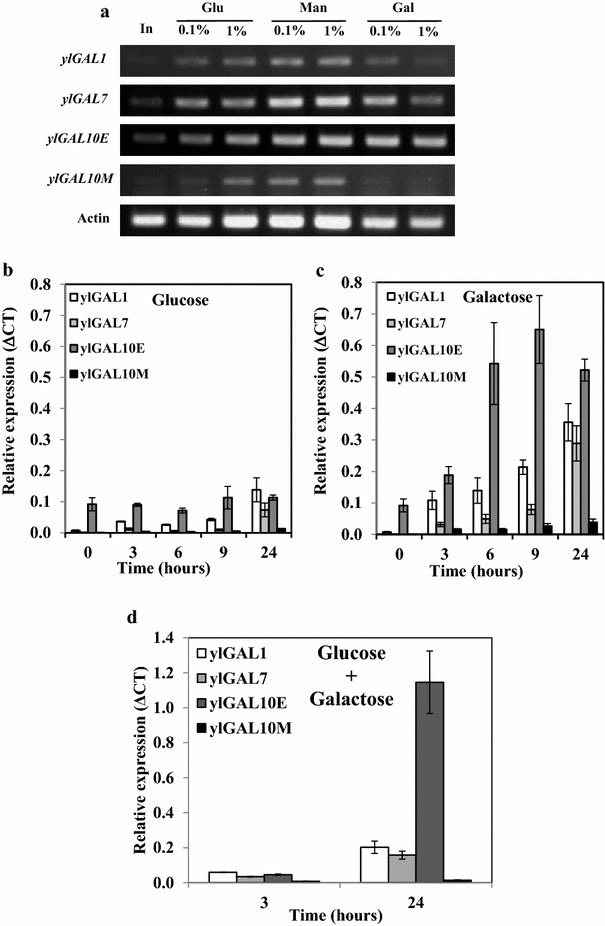
Expression profiles of *ylGAL* genes in *Y. lipolytica* W29. Cells were first growing for 16 h in YNB medium with 1.0 % of glucose and then incubated for 3–24 h in YNB medium containing the indicated sugar at 0.1 or 1.0 %. End-point RT-PCR was performed after 3 h incubation in glucose, mannose, or galactose (**a**). Kinetics of *ylGAL* genes expression was monitored by quantitative RT-PCR in *Y. lipolytica* W29 incubated in YNB medium containing 1.0 % glucose (**b**), 1.0 % galactose (**c**), or a mixture of 1.0 % glucose and 1.0 % galactose (**d**). Gene expression levels were normalized based on the expression levels of the actin gene (Δ*C*_T_). *In* inoculum

To examine *ylGAL* gene expression, *Y. lipolytica* was grown in 1.0 % glucose medium followed by its transfer to a suite of media containing two different concentrations (0.1 and 1.0 %) of three different monosaccharides (galactose, glucose, and mannose), and transcription analyses were performed (Fig. 2a; for details see “Methods”). Galactose was the target compound, glucose was a potential repressor, and mannose was a neutral sugar in catabolite repression. The three main *ylGAL* genes (*ylGAL1, ylGAL7, ylGAL10E*) were expressed under all the conditions, but *ylGAL10M*, the least important gene in galactose utilization, showed very weak expression (Fig. 2a). This pattern is similar to that observed in *H. jecorina* [17, 19]. Because the *ylGAL* genes were expressed when *Y. lipolytica* was grown on glucose, it appears that they are not subject to catabolic repression as they are in *S. cerevisiae* [12, 13]. A similar pattern was observed in *A. niger*, which shows poor growth on galactose, even though all of its Leloir orthologs are expressed on all the carbon sources studied [20].

Since the *ylGAL* genes were not repressed by glucose, a kinetics analysis of their quantitative expression on glucose, galactose and mixture of both was conducted (Fig. 2b–d). The gene profiles show that, on glucose, expression of the UDP-glucose-4-epimerase gene (*ylGAL10E*) was constant, whereas expression of galactokinase (*ylGAL1*) and galactose-1-phosphate uridyl transferase (*ylGAL7*) genes increased after 24 h (Fig. 2b). Once again, the galactose mutarotase gene (*ylGAL10M*) had the lowest expression levels. On galactose, there was a slight increase in expression of *ylGAL* genes (*ylGAL1*, *ylGAL7*, *ylGAL10E*) over time: it reached between 2.7 and 4.6 after 24 h (Fig. 2c). Therefore, the *ylGAL* genes were upregulated in the presence of galactose. The expression of *ylGAL* genes in the mixture of 1.0 % glucose and 1.0 % galactose (Fig. 2d) resembled the expression profile of these genes on 1.0 % glucose (Fig. 2b), except for *ylGAL10E* gene which was more than 10 times upregulated by the presence of galactose in the medium (Fig. 2d). This result indicates that in *Y. lipolytica*, the *ylGAL* genes are not subject of glucose catabolite repression since *ylGAL10E* can be upregulated by galactose also in the presence of glucose. In *A. nidulans* and *H. jecorina*, galD (GAL7) and galE (GAL1) have also been reported to be upregulated in the presence of galactose versus glucose [17, 19, 21]. However, in contrast to *ylGAL10E* gene, which is also upregulated by galactose, the expression of its homolog *GAL10* of *H. jecorina* is not improved by this sugar [16, 18].

### *Yarrowia lipolytica* GAL genes are functional

**Fig. 3:**
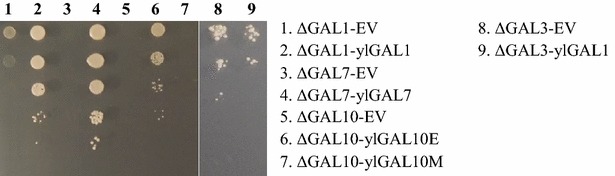
Complementation of *GAL* gene deletion in *S. cerevisiae* using GAL homologs found in *Y. lipolytica*. EV—strains complemented for uracil deletion using a pRS426TEF empty vector

To investigate the activity of the proteins encoded by the *Y. lipolytica* GAL genes, a functional complementation test was performed using *S. cerevisiae*. The *ylGAL* genes were amplified and cloned into a multicopy pRS426 vector under the control of the scTEF promoter. The *S. cerevisiae* transformants obtained were subsequently tested to determine if they could grow when galactose was the sole carbon source (Fig. 3). The three cloned *ylGAL* genes, *ylGAL1*, *ylGAL7*, and *ylGAL10E*, complemented their corresponding *S. cerevisiae* GAL-deletion mutants and restored growth on galactose. This result shows that the *ylGAL* genes encode functional Leloir proteins. The fact that the *S. cerevisiae* mutant (depleted for both epimerase and mutarotase functions) expressing only the *ylGAL10E* gene demonstrated growth, while the mutant expressing the *ylGAL10M* gene did not confirm what Seiboth et al. [18] found: that the epimerase, not the mutarotase, activity of *scGAL10* is required for *S. cerevisiae* to be able to use galactose as a sole carbon source [11]. Because *K. lactis* galactokinase demonstrates two activities—it acts as an enzyme and a regulatory protein [8]—the putative regulatory function of *Y. lipolytica* galactokinase was also examined. The *ylGAL1p* gene, which complemented the *S. cerevisiae* ΔGAL1 mutant, was not able to restore normal growth on galactose for the *S. cerevisiae* ΔGAL3 mutant (Fig. 3), in contrast to its *K. lactis* counterpart [8]. *ScGAL3*, which is an ohnolog of *scGAL1*, diverged after a whole genome duplication event, losing the ancestral gene’s enzymatic activity but retaining its regulatory function. This finding suggests that *ylGAL1* does not share *scGAL3*’s regulatory role; this function was either lost in *Y. lipolytica* over the course of evolution, or acquired later in hemiascomycetes (perhaps at the base of the Saccharomycetaceae branch).

### Validation of Leloir pathway functionality in *Yarrowia lipolytica*

**Fig. 4:**
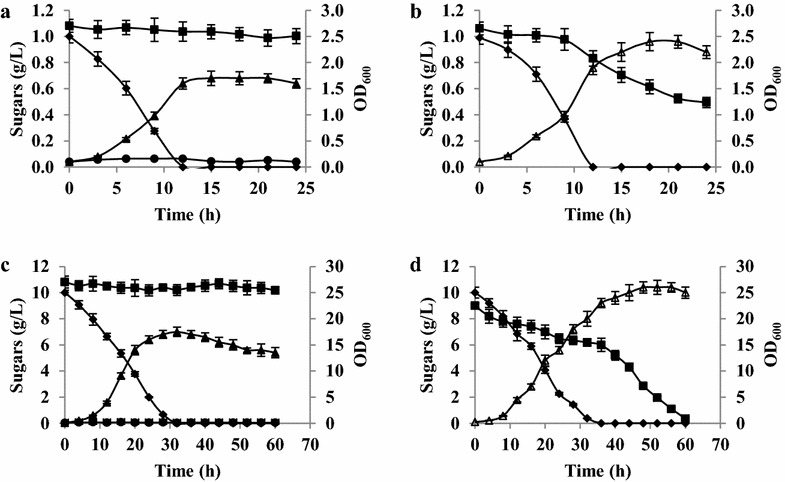
Changes in concentration of sugars and growth during cultures of *Y. lipolytica* W29 in YNB medium with glucose and galactose. **a** YNB medium with single 0.1 % glucose or 0.1 % galactose, **b** YNB medium with mixture of 0.1 % glucose and 0.1 % galactose, **c** YNB medium with single 1 % glucose or 1 % galactose, **d** YNB medium with mixture of 1 % glucose and 1 % galactose. Glucose (*filled diamond*), galactose (*filled square*), optical density of cells growing in cultures with—glucose (*filled triangle*), galactose (*filled circle*), mixture of glucose and galactose (*unfilled* *triangle*)

Given the evidence that the Leloir pathway was potentially functional in *Y. lipolytica,* galactose utilization by the species was investigated (Fig. 4). The *ylGAL* genes were expressed when the yeast was grown on glucose, but galactose alone did not allow growth. As a result, growth and sugar consumption by *Y. lipolytica* W29 (the wild-type strain) in YNB medium containing a mixture of glucose and galactose (0.1 or 1.0 %) were analyzed. First, a control experiment was conducted in which growth and sugar consumption on glucose versus galactose were examined. As expected, when galactose was the sole carbon source, it was not consumed, whereas 0.1 and 1.0 % glucose were depleted within 12 and 32 h, respectively (Fig. 4a, c). Surprisingly, when the same strain was grown in medium containing both 0.1 % glucose and 0.1 % galactose, it was able to use both sugars (Fig. 4b). The specific glucose utilization rate was the same as for the glucose-only culture (0.60 ± 0.04 g/g/h). In contrast, the specific galactose utilization rate reached ten times lower value than the one for glucose (0.060 ± 0.005 g/g/h) and remained constant through the process. In addition, sugar consumption was also tested using cultures containing higher concentrations of both monosaccharides (1 %) to eliminate the influence of any putative sugar sensors, which could change carbon metabolism (Fig. 4d). As in the previous experiment, *Y. lipolytica* W29 used both glucose and galactose from the beginning, but the glucose consumption rate was much higher than the galactose consumption rate (0.15 ± 0.01 g/g/h versus 0.030 ± 0.003 g/g/h, respectively). It is worth noting that, not only was galactose consumed by the cells, but it was also partially used for biomass production (Fig. 4b, d). In addition, deletion of *ylGAL10E* gene resulted in dramatic decrease of galactose consumption (Additional file 1), stating the involvement of the Leloir pathway in galactose utilization.

**Fig. 5:**
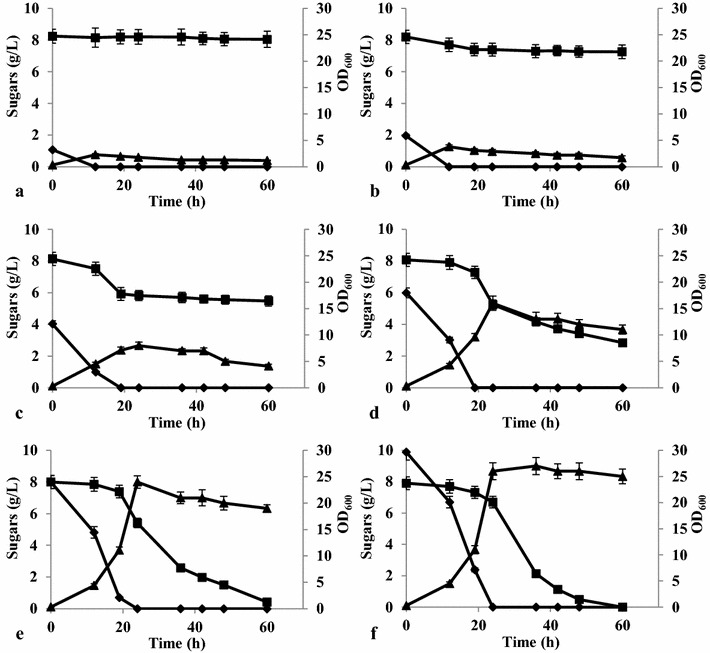
Comparison of growth and sugar utilization of *Y. lipolytica* W29 in YNB medium with 1 % galactose and different concentration of glucose: 0.1 % (**a**); 0.2 % (**b**); 0.4 % (**c**); 0.6 % (**d**); 0.8 % (**e**); 1.0 % (**f**). Glucose (*filled diamond*), galactose (*filled square*), OD_600_ (*filled triangle*)

The next step was therefore to determine the concentration of glucose that *Y. lipolytica* requires to be able to use galactose. *Y. lipolytica* W29 was therefore grown in a suite of media containing 1.0 % galactose and a range of glucose, from 0.1 to 1.0 %. At a glucose concentration of 0.1 %, growth was very weak and galactose was not consumed (Fig. 5a). At higher glucose concentrations (0.2–0.4 %), a small amount of galactose was also utilized; however, after the glucose was used up, galactose consumption stopped (Fig. 5b, c). At even higher glucose concentrations, *Y. lipolytica* consumed galactose more efficiently, following a short delay (Fig. 5d–f). Galactose consumption increased once the glucose had been drained from the medium, which is also when the cells enter into the stationary phase (Fig. 5d–f). The fact that *Y. lipolytica* needs glucose concentrations to be higher than 0.4 % to efficiently use galactose suggests that it first requires access to a metabolically favorable carbon source that promotes growth and viability before it can use galactose. This phenomenon could result because *Y. lipolytica* is strictly aerobic, and the large amount of energy required to synthesize Leloir proteins cannot be acquired when the yeast begins its growth on galactose. In *S. cerevisiae* exposed to oxygen-limited conditions, when the yeast is switched from glucose to galactose, energy levels rapidly drop, and Leloir proteins are not synthesized in sufficient quantities to make galactose utilization possible [25]. However, this explanation for the observed *Y. lipolytica* phenotype must be investigated further.

### Overexpression of all *Yarrowia lipolytica* GAL genes induces efficient growth on galactose

**Fig. 6:**
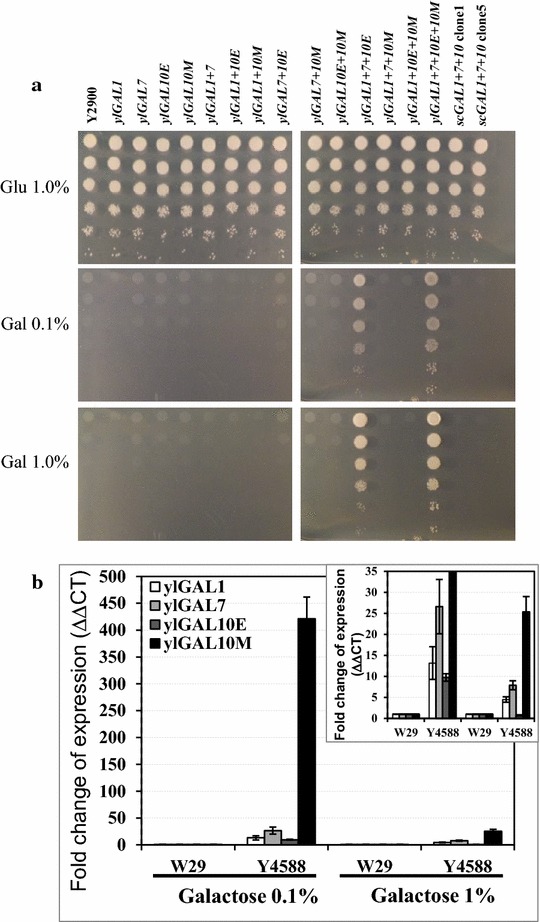
Functional analysis of Leloir pathway overexpression in *Y. lipolytica* and fold change in the expression of the *ylGAL* genes. Growth of different *Y. lipolytica* transformants overexpressing *ylGAL* and *scGAL* genes on plates of YNB medium containing glucose or galactose—incubation at 28 °C, 48 h (**a**). Fold changes in the expression of *ylGAL* genes in the quadruple mutant, Y4588 (in which all the Leloir genes were overexpressed), relative to *Y. lipolytica* W29 (the wild-type strain) (**b**); yeast were incubated for 3 h in YNB medium containing 0.1 or 1.0 % galactose. Gene expression levels were normalized based on the expression of the actin gene

Because *Y. lipolytica*’s GAL genes were found to be functional, the goal became to see if it was possible to improve the Leloir pathway such that *Y. lipolytica* could efficiently use galactose as a sole carbon source. All the *ylGAL* genes were placed under the control of the strong constitutive TEF promoter and introduced individually or in various combinations into the *Y. lipolytica* PO1d background. The resulting strains were then tested to see if they could grow on galactose alone. When the transformants were grown on glucose, they showed normal growth and no morphological changes, suggesting that their physiology was not affected (Fig. 6a). Only mutants in which *ylGAL1*, *ylGAL7*, and *ylGAL10E* were simultaneously overexpressed could grow within 48 h on both, 0.1 and 1.0 % galactose (Fig. 6a, line 12). Mutarotase activity, which is not required for galactose metabolism, nonetheless appeared to slightly improve galactose utilization (Fig. 6a, line 15). Interestingly, the overexpression of all the *S. cerevisiae* Leloir genes in *Y. lipolytica* was not efficient and resulted in weak, delayed growth (Additional file 2A). Additional analysis of growth patterns showed that the *Y. lipolytica* transformants overexpressing the *ylGAL* genes, with the exception of Y4585 and Y4588, took a long time to grow on galactose. The Y4577 strain, in which *ylGAL1* and *ylGAL7* were overexpressed, showed visible growth after 2 weeks of incubation but revealed normal morphology on plates (Additional file 2B). This fact supports the observation, that the native *ylGAL10E* expression is higher than that of the rest of the *ylGAL* genes, but needs time for it to reach an efficient level. In addition, the Y4578 strain, in which *ylGAL1* and *ylGAL10E* were overexpressed, also showed visible growth after 2 weeks of incubation, albeit much less efficient than Y4577 (Additional file 2B). This finding underscores that *ylGAL7* expression results in functional protein biosynthesis but not normal levels for galactose utilization in the whole cell population.

The strong increase in gene expression in the quadruple mutant (Y4588) showed that, when galactose concentrations were low, each gene was overexpressed compared to the parental strain; for instance, expression was 400 times higher for *ylGAL10M* (Fig. 6b). Surprisingly, when galactose concentrations were high (1.0 %), the change in gene expression levels was not so sharp, especially in the case of *ylGAL10E.* The parental strain expresses *ylGAL10E* at high levels when grown in 1.0 % galactose, which could explain why the overexpression of this particular gene did not increase transcript levels (Fig. 6b). Overexpressing all four of the *ylGAL* genes efficiently activated the Leloir pathway and created a strain that can use galactose as its sole carbon source.

### Sugar utilization kinetics and transporters’ expression in *Yarrowia lipolytica* overexpressing *ylGAL* genes

**Fig. 7:**
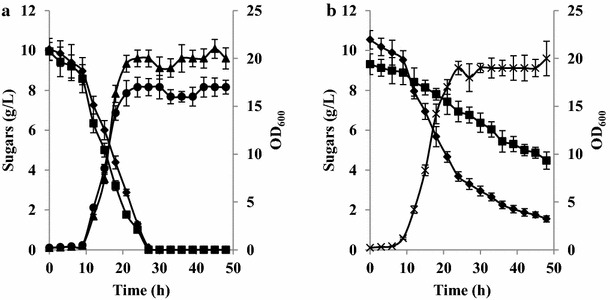
*Yarrowia lipolytica* Y4588 growth and sugar consumption over 48 h in YNB medium containing only 1 % glucose, only 1 % galactose (**a**), or a mixture of both 1 % glucose and 1 % galactose (**b**). Glucose (*filled diamond*), galactose (*filled square*), OD_600_ in glucose (*filled triangle*), OD_600_ in galactose (*filled circle*), and OD_600_ in the mixture of both sugars (*x*)

Glucose must be present in the culture medium for the *Y. lipolytica* wild-type (W29) strain to be able to utilize galactose. Overexpression of all the genes in the Leloir pathway allowed *Y. lipolytica* cells to grow on media whose sole carbon source was galactose. The next step was to determine if glucose had an effect on galactose utilization in the *Y. lipolytica* quadruple mutant (Y4588). To this end, its growth and sugar utilization in 1 % glucose, 1 % galactose, and a mixture of 1 % glucose and 1 % galactose were characterized. The kinetics of sugar consumption were the same when the strain was grown in the single sugar cultures, reaching a specific consumption rate of 0.13–14 ± 0.01 g/g/h; slightly more biomass was produced when the strain was grown on glucose alone (Fig. 7a). However, sugar utilization dynamics changed in the mixed medium (Fig. 7b). During the exponential growth phase, the specific glucose and galactose utilization rates were 0.13 ± 0.01 and 0.040 ± 0.003 g/g/h, respectively, while during the stationary phase, both values decreased to 0.020 ± 0.001 and 0.030 ± 0.003 g/g/h for glucose and galactose, respectively. This same pattern was also seen at lower concentrations of both sugars (0.1 and 0.5 %, respectively; data not shown). Overall, growth and sugar consumption were the same on the glucose-only and mixed-sugar media; for instance, at 18 h of incubation, consumption was around 5.5–6 g/L for both (Fig. 7). Even though the galactose utilization pathway had clearly been activated, glucose was obviously the preferred carbon source.

The transport of galactose inside the cell is the first step in the metabolic process. It may be modified in the quadruple mutant or may be affected by the presence of glucose. In *Y. lipolytica*, six genes have been identified as hexose transporters from a HXT-like family; they have been named in series, YHT1 to YHT6, and have been reported to transport galactose (Lazar et al., in preparation; [26]). The uptake efficiency of both galactose and glucose was compared in *S. cerevisiae* hxt null mutants transformed with each of these six genes (Additional file 3A). Four of the transporters, YHT1–YHT4, allowed significant galactose or glucose consumption; uptake efficiency, however, varied (Additional file 3A). Only two of the transporters, YHT1 and YHT4, were expressed at high levels in *Y. lipolytica* Y4588 strain grown in either 1 % glucose or 1 % galactose, similarly as in the W29 (WT) strain (Additional file 3B). These findings suggest that this set of galactose transporters is affected neither by the genetic background (WT or Y4588) nor by the carbon source. Because galactose consumption was much slower in the presence of glucose, it can be hypothesized that galactose transport was somehow inhibited due to competition with glucose, even if their expression was not repressed. However, potential interactions between glucose metabolism and galactose metabolism cannot be excluded.

### Galactose is a good substrate for industrial processes using *Yarrowia lipolytica*

To confirm that galactose could be effectively used in industrial applications (e.g., the *Y. lipolytica* Y4588 strain could efficiently produce citric acid and lipids), batch cultures were raised in flasks and bioreactors. All the biomass, citric acid, and lipid production results are summarized in Table 2.

**Table 2:** Biomass, citric acid, and lipid production

Parameter		*X* (g/L)	*Y*_X/S_ (g/g)	CA (g/L)	*Y*_CA/S_ (g/g)	*Y*_CA/X_ (g/g)	FA (g/L)	*Y*_FA/S_ (g/g)	*Y*_FA/X_ (g/g)
Flasks									
Glu 6 % (C/N 60)	W29	11.15	0.24	4.18	0.09	0.37	1.92	0.041	0.172
	Y4588	10.90	0.21	18.17	0.35	1.67	1.87	0.036	0.172
Gal 6 % (C/N 60)		11.27	0.19	12.58	0.22	1.12	1.98	0.034	0.176
Gal 6 % (C/N 100)		8.45	0.17	16.96	0.35	2.01	1.79	0.037	0.212
Bioreactors									
Gal 6 % (C/N 100)	Y4588	19.4	0.34	29.2	0.51	1.51	3.22	0.056	0.166

*Y. lipolytica* W29 and Y4588 strains were grown for 96 h in YNB medium containing 6 % glucose or 6 % galactose in flasks and bioreactors

In this analysis, SD did not exceed 7.5 %

*X* dry biomass, *FA* fatty acids, *CA* citric acid, *Y*_*X/S*_ yield of biomass from substrate consumed, *Y*_*CA/S*_ yield of citric acid from substrate consumed, *Y*_*CA/X*_ yield of citric acid from dry biomass, *Y*_*FA/S*_ yield of fatty acids from substrate consumed, *Y*_*FA/X*_ yield of fatty acids from dry biomass

The *Y. lipolytica* wild-type (W29) and quadruple mutant (Y4588) strains produced similar amounts of biomass (~11 g/L) when grown in both glucose and galactose media with C/N ratio of 60. Biomass yield ranged from 0.19 to 0.24 g/g. When the C/N ratio of the galactose medium was increased to 100, biomass yield decreased slightly (11 %).

Interestingly, in glucose, citric acid biosynthesis and yield were around four times higher for Y4588 than for W29. Furthermore, Y4588 was able to produce three times more citric acid from galactose (C/N 60) than did W29 grown in glucose. Finally, when the C/N ratio was increased to 100, biomass and citric acid yield from galactose increased by 55 and 37 %, respectively.

Galactose could also be used to efficiently produce lipids. In glucose with a C/N ratio of 60, W29 and Y4588 produced similar amounts of total lipids. Y4588 was able to produce comparable amounts of total lipids in galactose- and glucose-only media (Table 2). Lipid yields for W29 and Y4588 ranged between 0.034 and 0.041 g/g. In addition, increasing the C/N ratio to 100 improved the lipid accumulation by 20 %; it reached 0.212 g/g. W29 had similar or lower yields when grown in glucose or fructose media with the same C/N ratio [2].

To study Y4588’s ability to produce citric acid and lipids in large quantities, the process was scaled up production by using 2-L bioreactors; this approach was informed by previous results obtained for galactose flask cultures (C/N ratio of 100). Overall, biomass, citric acid, and lipid production were dramatically improved (Table 2). Up to 19.4 g/L of biomass was produced under these conditions, representing an increase of 100 %. Citric acid production reached 29.2 g/L and thus the conversion of substrate to citric acid was improved by 31 %. In addition, the lipid production increased from 1.79 to 3.22 g/L, which signifies that 34 % more galactose was converted into lipids. The construction of a *Y. lipolytica* strain that is able to produce citric acid and lipids from galactose is a very important step in bypassing issues related to the use of food-based substrates in industrial applications. In addition, it will be important to activate the Leloir pathway in a strain optimized for lipid accumulation to reach as high titer of biolipids from galactose as recently reported by Qiao et al. from glucose [27]. Activation of the Leloir pathway in a strain described recently by Lazar et al. [2] is currently in progress.

## Conclusions

Galactose is potentially a useful alternative source of carbon for microbiological industrial processes. Our research on the Leloir pathway of galactose utilization in *Y. lipolytica* has revealed that this yeast is more similar to filamentous fungi such as *H. jecorina*, *A. nidulans*, and *A. niger* than to *S. cerevisiae* and *K. lactis* in terms of its genetic organization and gene regulation. The *ylGAL* genes are not repressed by glucose and are upregulated by galactose. They encode fully functional proteins. Hexose transporters genes involved in galactose transport are expressed on both, glucose and galactose: however, galactose transport is likely inhibited by substrate competition. In addition, *ylGAL* genes are not expressed enough in wild-type *Y. lipolytica* to allow growth on galactose. A very efficient galactose-metabolizing *Y. lipolytica* strain was created by fully activating the Leloir pathway, making it possible to use this sugar as a substrate in biotechnological applications. This yeast can be successfully used in citric acid and lipid production; making these processes more effective is currently the focus of intensive research worldwide. The use of galactose as an industrial substrate is especially appealing because it is a component of hemicelluloses and is also found in pectins. Consequently, its metabolism by genetically modified microbes may make it possible to efficiently exploit lignocellulosic biomass for biotechnological purposes, reducing the need to use substrates that are already in demand for food production.

## Methods

### Yeast strains and plasmids

**Fig. 8:**
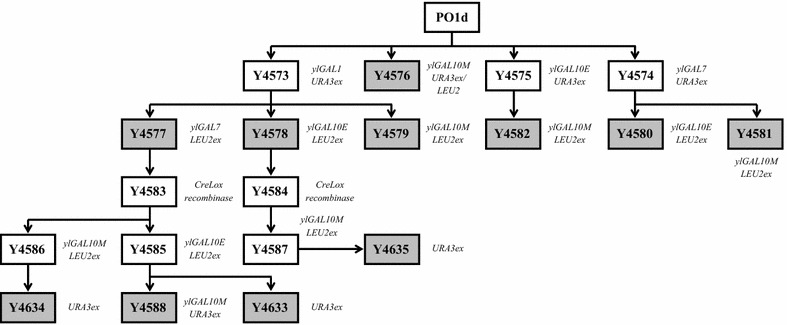
Construction of *Y. lipolytica* strains overexpressing *ylGAL* genes. The auxotrophic PO1d strain was used as the acceptor strain. The genes were inserted one by one to create strains that overexpressed different combinations of the *ylGAL* genes; URA3ex and LEU2ex were used as selection markers. To recover prototrophy in some of the *Y. lipolytica* strains (*gray squares*), cassettes containing the URA3 excisable marker or the purified *Sal*I fragment of a pINA62 plasmid that contained the *LEU2* gene were inserted into the transformants’ genomes [28]. In turn, to recover URA3 and LEU2 auxotrophy, which was necessary to continue the transformation process, a JME547 plasmid containing Cre-Lox recombinase was used [35]

The strains and plasmids used in this study are listed in Additional file 4. All the *Y. lipolytica* transformants derived from the genetic background of the W29 wild-type strain and of the derivative auxotrophic strain PO1d (Ura^−^Leu^−^) [28]. Because many strains of various kinds overexpressing *Y. lipolytica* GAL genes were created, only the—quadruple overexpressing strain, Y4588, which overexpressed all four of the *ylGAL* genes, will be described in detail here. Construction of the other strains is depicted in Fig. 8. Plasmids containing *ylGAL* genes were constructed using URA3ex and LEU2ex selection markers. Strain Y4573, which overexpressed the *Y. lipolyticaylGAL1* gene (YALI0C13090g), was obtained by introducing the overexpression cassette from JME2542 containing the URA3ex selection marker into PO1d. Subsequently, the cassette containing the *ylGAL7* gene (YALI0F23947g) and a LEU2ex marker taken from JME2547 was introduced into the Y4573 strain, thus generating the prototrophic Y4577 strain. To excise both selection markers, Y4577 was transformed with JME547 plasmid containing Cre-Lox recombinase and hygromycin selection. This process generated the auxotrophic Y4583 strain (Ura^−^Leu^−^). Immediately afterward, the *ylGAL10E* gene (YALI0E26829g) overexpression cassette containing the LEU2ex marker from a JME2548 plasmid was introduced into the Y4583. Then, the cassette containing the *ylGAL10M* gene (YALI0C09570g) and the URA3ex marker taken from a JME2545 plasmid was introduced, generating Y4588, which overexpressed the entire Leloir pathway.

The strain *Y. lipolytica* YLZ68 was obtained by transformation of PO1d strain with NotI digested fragment of ZLE19 vector for *ylGAL10E* gene deletion with URA3ex as selection marker. To recover prototrophy, a purified *Sal*I fragment of the pINA62 plasmid that contained the *LEU2* gene was introduced [29].

*Yarrowia lipolytica* strains expressing *S. cerevisiae* GAL genes were constructed as depicted in Additional file 5. All the plasmids were prepared as described above.

Functional complementation analysis of the *Y. lipolytica* GAL genes was performed using *S. cerevisiae* strains from which the GAL genes had been deleted. The *Y*. *lipolytica* galactokinase (*ylGAL1*) gene was introduced into *S. cerevisiae* Δgal1 (Y4475) as well as into the Δgal3 (Y4846) strains using JME2735 plasmid, thus generating the Y4595 and Y4875 strains, respectively. To complement the Δgal7 strain, a JME2736 plasmid (*ylGAL7*) was introduced into Y4473, generating Y4596. Finally, Δgal10 was complemented separately by the *ylGAL10E* and *ylGAL10M* genes, which was accomplished by introducing JME2738 and JME2739 plasmids into the Y4474 strain, yielding Y4597 and Y4690, respectively. All the *S. cerevisiae* deletion mutants—the control strains—were also complemented for uracil auxotrophy by the insertion of an empty pRS426TEF vector.

All the genes introduced into *Y. lipolytica* and *S. cerevisiae* were under the control of the constitutive *TEF* promoter [30]. Yeast transformation was performed using the lithium acetate procedure [31]. Expression cassettes were integrated into the genome of *Y. lipolytica* [32] or were in the replicative pRS426TEF vector in *S. cerevisiae* [30].

### General genetic techniques and plasmid construction

Standard molecular genetics techniques were used throughout this study [33]. Restriction enzymes were obtained from New England Biolabs (Ipswich, England). Genomic DNA from the transformants was prepared using phenol:chlorophorm extraction method. PCR amplification was performed using an Eppendorf 2720 thermal cycler; both Taq (Promega, Madison, WI) and Pfu (Stratagene, La Jolla, CA) DNA polymerases were employed. PCR fragments were then purified using a QIAgen Purification Kit (Qiagen, Hilden, Germany), and DNA fragments were recovered from the agarose gels using a QIAquick Gel Extraction Kit (Qiagen, Hilden, Germany). The Staden software package was used for gene sequence analysis [34]. The GAL genes were amplified using the primers listed in Additional file 6. The restriction sites in the primer sequences enabled the genes to be cloned into JME1128 plasmids that had been digested with *Bam*HI-*Avr*II, as previously described [35]. Auxotrophies were restored via excision using the Cre-lox recombinase system as described previously [36].

The deletion cassette was typically generated by PCR amplification according to Fickers et al. [36]. First, the promoter (P) and terminator (T) regions were amplified from *Y. lipolytica* W29 genomic DNA as the template and with the gene-specific P1/P2 and T1/T2 oligonucleotides as primer pairs (Additional file 6). Primers P2 and T1 contained an extension to introduce the I-*sce*I restriction site.

For the *ylGAL10E* gene, primer pairs ylGAl10E-P1/ylGAL10E-P2 and ylGAL10E-T1/ylGAL10E-T2 were used (Additional file 6). The P and T regions were purified and used for the second PCR. The resulting PT fragment was ligated into pCR4Blunt-TOPO. The *URA3* marker was then introduced at the *I*-*sce*I site, yielding the construct ZLE19 containing the *ylGAL10E*-*PUT* cassette.

To complement the *S. cerevisiae* strains GAL genes of which had been deleted, the corresponding genes were cloned in a pRS426 vector using the constitutive TEF promoter and the uracil selection marker. The corresponding *ylGAL* genes were amplified and digested, as described above, with the restriction enzymes listed in Additional file 6.

### Growth media

Media and growth conditions for *Escherichia coli* were identical to those as described by Sambrook and Russell [33], as were those of *Y. lipolytica* [28]. Rich (YPD) medium was prepared using 20 g/L Bacto™ Peptone (Difco, Paris, France), 10 g/L yeast extract (Difco), and 20 g/L glucose (Merck, Fontenay-sous-Bois, France). Minimal (YNB) medium was prepared using 1.7 g/L yeast nitrogen base (without amino acids and ammonium sulfate, Difco), 10 g/L glucose (Merck), 5 g/L NH_4_Cl, and 50 mM phosphate buffer (pH 6.8). To complement the auxotrophies, 0.1 g/L uracil or leucine (Difco, Paris, France) was added as necessary.

### Growth in microtiter plates

Precultures were obtained from frozen stock, inoculated into tubes containing 5 mL YPD medium, and cultured overnight (170 rpm, 28 °C). They were then washed with sterile distilled water; cell suspensions were adjusted to an OD_600_ of 0.1. Yeast strains were grown in 96-well plates in 200 µL of minimal YNB medium containing 10 g/L of either glucose or galactose. Culturing was repeated three times; 2–3 technical replicates were performed for each condition. Cultures were maintained at 28 °C under constant agitation using a Biotek Synergy MX microtiter plate reader (Biotek Instruments, Colmar, France); each culture’s optical density at 600 nm was measured every 20 min for 72 h.

### Growth and media used in the experiments

Initial precultures were established by inoculating 50 mL of YPD medium in 250-mL Erlenmeyer flasks with the yeast strains; this was followed by an overnight shaking step at 28 °C and 170 rpm. The cell suspensions were washed three times with sterile distilled water and used to inoculate 50 mL of YNB medium to an OD_600_ of 0.25. The cultures were grown until all the available sugar had been consumed.

In the galactose utilization experiment, the YNB medium contained one of the following: 1.0 g/L of glucose; 1.0 g/L of galactose; 1.0 g/L of both glucose and galactose; or 10.0 g/L of both glucose and galactose. In addition, galactose utilization was observed in YNB medium containing 10.0 g/L of galactose and either 1.0; 2.0; 4.0; 6.0; 8.0; or 10.0 g/L of glucose.

### Analysis of growth on galactose by drop test

To analyze the different strains’ growth on galactose, a drop test was performed on cultures grown on YNB plates. The *Y. lipolytica* strains were grown in 5 mL of YPD medium for 24 h. The cell suspensions were then washed twice with water and re-suspended at an OD_600_ of 1. Successive tenfold dilutions were performed (10^0^–10^−5^), and 5 µl of each dilution were spotted onto YNB plates containing 10.0 g/L of glucose, 1.0 g/L of galactose, or 10.0 g/L of galactose. Pictures were taken after the cultures had been incubated at 28 °C for 48 h. The same protocol was used for the *S. cerevisiae* strains. However, the YNB media on which they were grown contained the following: 10.0 g/L of either glucose or galactose; 6.5 g/L of YNB; 10.0 g/L of (NH_4_)_2_SO_4_; 0.018 g/L of leucin; 0.0115 g/L of histidin; and 0.025 g/L of lysin.

### Analysis of galactose uptake by *Yarrowia lipolytica* hexose transporters

To analyze galactose uptake, *Y. lipolytica* hexose transporters were expressed one at a time in the *S. cerevisiae* hxt null mutant strain EBY.VW4000 (kindly provided by E. Boles, Goethe University, Frankfurt am Main, Germany) using a replicative pRS426 vector containing the TEF promoter [Lazar et al., in preparation]. The cells were grown in 5 mL of YNB medium containing 20 g/L of maltose; the medium was refreshed 3 times per 24-h period to increase 2μ plasmid copy number. The cell suspensions were washed three times with sterile distilled water and used to inoculate 50 mL of YNB medium that contained either 10.0 g/L of glucose or 10.0 g/L of galactose. The culture conditions were as described above ("Growth and media used in the experiments"). OD_600_ and sugar concentration were analyzed.

### Conditions and media used for lipid biosynthesis from galactose in flasks and bioreactors

The precultures were prepared as described above ("Growth and media used in the experiments"). The main culture was grown on 50 mL of YNB medium (C/N 60) containing the following: galactose 60.0 g; YNB 1.7 g; NH_4_Cl 1.5 g; 0.7 g KH_2_PO_4_, and 1.0 g MgSO_4_ × 7H_2_O in 1 L. The pH was kept at 6.8 using 0.05 M phosphate buffer. Tap water was used as a source of microelements. To increase the C/N ratio of the medium to 100, 1.25 g/L of NH_4_Cl was used. Culture conditions were as described above ("Growth and media used in the experiments").

Lipid biosynthesis was also evaluated using batch cultures (BC) that were kept in 5-L stirred-tank BIO-STAT B-PLUS bioreactors (Sartorius, Frankfurt, Germany) for 96 h under the following conditions: 2-L working volume, 28 °C, 800 rpm of agitation, and 3.5-L/min aeration rate. The production medium was prepared as described above. The pH was kept at 6.8 using a 40 % (w/v) NaOH solution. The cultures were grown in 0.2 L of YPD medium in 0.5-L flasks at 170 rpm, at 28 °C for 48 h. The volume of the inocula added to the bioreactor cultures was equal to 10 % of the total working volume.

### Analysis of *ylGAL* gene and hexose transporter expression

For the expression experiments, the wild-type strain (W29) and the quadruple mutant (Y4588) were grown at 28 °C in YNB medium that had been supplemented with 10 g/L glucose. After 16 h, the cell suspensions were washed twice with distilled water and transferred into fresh YNB medium with various sugars. For single sugar experiment, the fresh medium contained either 1.0 or 10.0 g/L of glucose, galactose, or mannose. Samples were harvested at 3 h post inoculation; three replicates were obtained. For the kinetics experiments, the cells were transferred to 1.0 % glucose, 1.0 % galactose or mixture of both 1.0 % glucose and 1.0 % galactose. The samples were harvested at 3, 6, 9 and 24 h post inoculation; three replicates were obtained. All the samples were frozen in liquid nitrogen and stored at −80 °C.

Total RNA was extracted using the RNeasy Mini Kit (Qiagen, Hilden, Germany), and 1.5 μg of each sample was treated with DNase (Ambion, Life Technologies). cDNA was synthesized using the Maxima First Strand cDNA Synthesis Kit for RT-qPCR (Thermo Scientific). PCR reactions were performed using specific primers that targeted the 3′ end of the genes (Additional file 6). For the semiquantitative RT-PCR analyses, PCR was performed using the GoTaq DNA Polymerase Kit (Promega). For the quantitative RT-PCR analyses, gDNA dilutions were first tested for primer efficiency and then retained if their efficiency rating was higher than 90 %. Amplifications were carried out using the Sso Advanced Universal SYBR Green Supermix Kit (BIO-RAD). The following program was used: 98 °C for 3 min, followed by 40 cycles of 98 °C for 15 s, 58 °C for 30 s, and 72 °C for 30 s. Finally, melting curves were generated to confirm amplification specificity. Both Δ*C*_T_ and ΔΔ*C*_T_ methods were used to calculate relative expression levels; a constitutive gene, actin, was utilized as the reference control [37].

### Analytical techniques used in this study

Lipid bodies were stained with BodiPy^®^ Lipid Probe (2.5 mg/mL in ethanol; Invitrogen). Cell suspension samples (OD_600_ = 5) were incubated for 10 min at room temperature. Images were obtained using a Zeiss Axio Imager M2 microscope (Zeiss, Le Pecq, France) equipped with a 100× objective lens and Zeiss filter sets 45 and 46 for fluorescence microscopy. Axiovision 4.8 software (Zeiss, Le Pecq, France) was used for image acquisition.

The fatty acids were converted into methyl esters, analyzed by gas chromatograph (GC) on a Varian Factor Four vf-23 ms column and quantified using C17:0 (Sigma) as internal standard, as described previously [2]. Citric acid, glucose, fructose, and sucrose were identified and quantified by HPLC (UltiMate 3000, Dionex-Thermo Fisher Scientific, UK) using an Aminex HPX87H column coupled with UV (210 nm) and RI detectors. The column was eluted with 0.01 N H_2_SO_4_ at a flow rate of 0.6 mL/min at room temperature. Compounds were identified and quantified via comparisons to standards. Before undergoing HPLC analysis, the samples were filtered on membranes with a pore size of 0.45 μm.

To determine dry biomass, cell pellets from the 15-mL culture samples were washed twice with distilled water, filtered on the above membranes, and dried at 105 °C using a WPS 110S weight dryer (Radwag, Poznań, Poland) until a constant mass was reached.

## Additional files

10.1186/s13068-015-0370-4 Changes in concentration of glucose (♦) and galactose (■) during cultures of *Y. lipolytica* W29 (A) and YLZ68 (B) in YNB medium with mixture of 1 % of each sugar.
10.1186/s13068-015-0370-4 Overexpression of *scGAL* and *ylGAL* genes in *Y. lipolytica*. Growth of *Y. lipolytica* transformants containing *scGAL* genes (*scGAL*1,7,10) on YNB medium containing 1 % galactose (A); yeast were incubated for 1 week at 28 °C. Growth of *Y. lipolytica* transformants overexpressing different combinations of *ylGAL* genes on YNB medium containing 1 % galactose (B); yeast were incubated for 2 weeks at 28 °C.
10.1186/s13068-015-0370-4 Sugar consumption by *S. cerevisiae* null mutants expressing *Y. lipolytica* hexose transporters after 72 h of growth in YNB medium containing 1 % glucose (■) or 1 % galactose (■) (A). Expression profiles of *Y. lipolytica* genes encoding hexose transporters in the W29 and Y4588 strains (B). Yeast were incubated for 3 h in YNB medium containing 1.0 % glucose or 1.0 % galactose. The amplification of the PCR fragment in the genomic DNA served as a control for the primers’ efficiency. Abbreviations: In - inoculum.
10.1186/s13068-015-0370-4 Bacteria and yeast strains used in this study.
10.1186/s13068-015-0370-4 Construction of the *Y. lipolytica* strains in which the *scGAL* genes were overexpressed. The auxotrophic PO1d strain was used as the acceptor strain. The genes were inserted one by one to create strains that overexpressed different combination of the *scGAL* genes; URA3ex and LEU2ex were used as selection markers. To recover auxotrophies necessary to continuing the transformation process, a JME547 plasmid containing Cre-Lox recombinase was used to transform *Y. lipolytica* Y3683, thus generating strain Y3686 [35]. In turn, to recover prototrophy in the *Y. lipolytica* Y3687 strain, a purified *Sal*I fragment of the pINA62 plasmid that contained the *LEU2* gene was introduced [28].
10.1186/s13068-015-0370-4 List of primers used in this study.

## Abbreviations

Gal: galactose
Glu: glucose
Man: mannose
*C*_T_: threshold cycle
OD_600_: optical density (*λ* = 600 nm)
X: dry biomass
FA: fatty acids
CA: citric acid
*Y*_X/S_: yield of biomass from substrate consumed
*Y*_CA/S_: yield of citric acid from substrate consumed
*Y*_CA/X_: yield of citric acid from dry biomass
*Y*_FA/S_: yield of fatty acids from substrate consumed
*Y*_FA/X_: yield of fatty acids from dry biomass
