# Supplementary material for: Awakening the endogenous Leloir pathway for efficient galactose utilization by Yarrowia lipolytica
Source: Biotechnol Biofuels. 2015 Nov 25;8:185. doi: 10.1186/s13068-015-0370-4 (PMC4659199; doi:10.1186/s13068-015-0370-4)
Supplement: Supplementary file 1 — 10.1186/s13068-015-0370-4 Changes in concentration of glucose (♦) and galactose (■) during cultures of Y. lipolytica W29 (A) and YLZ68 (B) in YNB medium with mixture of 1 % of each sugar. [file 13068_2015_370_MOESM1_ESM.docx]

**Additional file 1.** Changes in concentration of glucose (♦) and galactose (■) during cultures of *Y. lipolytica* W29 (A) and YLZ68 (B) in YNB medium with mixture of 1% of each sugar.

**B**

**A**
