# Supplementary material for: Awakening the endogenous Leloir pathway for efficient galactose utilization by Yarrowia lipolytica
Source: Biotechnol Biofuels. 2015 Nov 25;8:185. doi: 10.1186/s13068-015-0370-4 (PMC4659199; doi:10.1186/s13068-015-0370-4)
Supplement: Supplementary file 2 — 10.1186/s13068-015-0370-4 Overexpression of scGAL and ylGAL genes in Y. lipolytica. Growth of Y. lipolytica transformants containing scGAL genes (scGAL1,7,10) on YNB medium containing 1 % galactose (A); yeast were incubated for 1 week at 28 °C. Growth of Y. lipolytica transformants overexpressing different combinations of ylGAL genes on YNB medium containing 1 % galactose (B); yeast were incubated for 2 weeks at 28 °C. [file 13068_2015_370_MOESM2_ESM.docx]

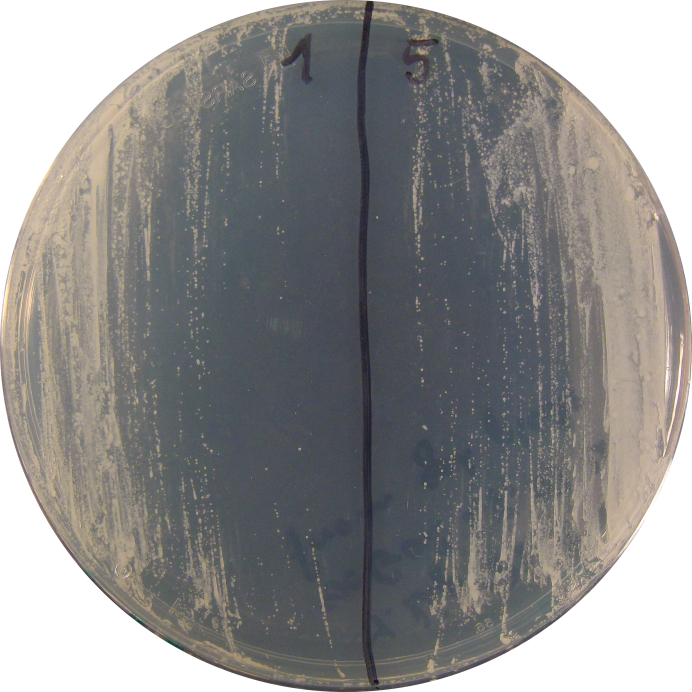


clone 1

clone 5

**A**

*ylGAL1+7*

*ylGAL1+10E* clone1

*ylGAL1+10E* clone2

*ylGAL7+10E*

**B**


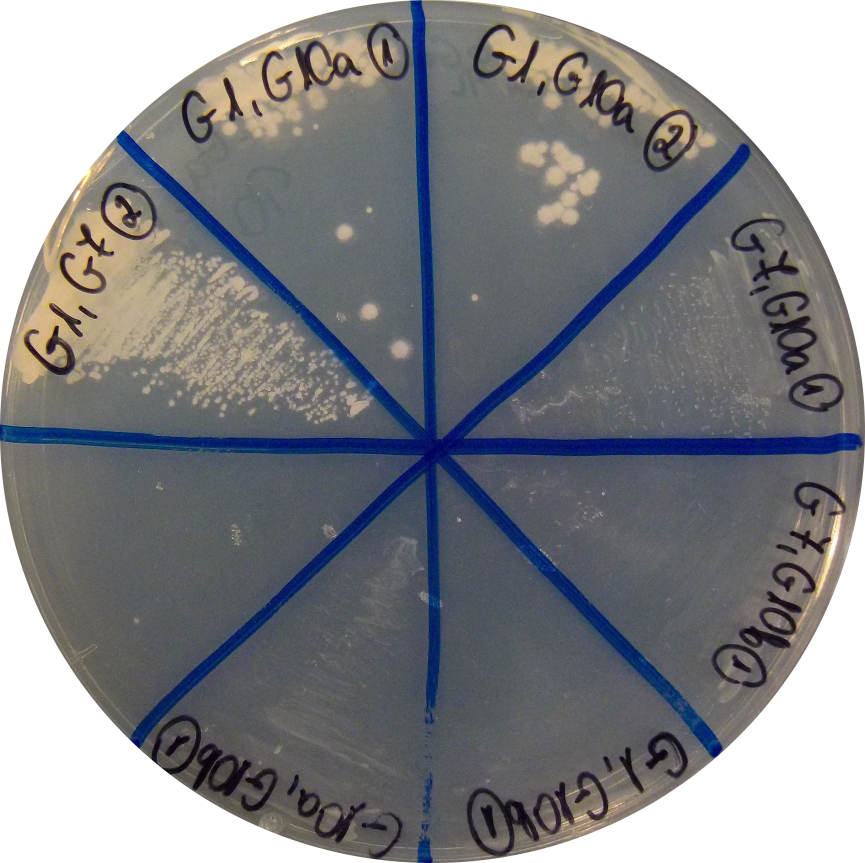


**Additional file 2.** Overexpression of *scGAL* and *ylGAL* genes in *Y. lipolytica*. Growth of *Y. lipolytica* transformants containing *scGAL* genes (*scGAL*1,7,10) on YNB medium containing 1% galactose (A); yeast were incubated for 1 week at 28ºC. Growth of *Y. lipolytica* transformants overexpressing different combinations of *ylGAL* genes on YNB medium containing 1% galactose (B); yeast were incubated for 2 weeks at 28ºC.
