# Supplementary material for: Awakening the endogenous Leloir pathway for efficient galactose utilization by Yarrowia lipolytica
Source: Biotechnol Biofuels. 2015 Nov 25;8:185. doi: 10.1186/s13068-015-0370-4 (PMC4659199; doi:10.1186/s13068-015-0370-4)
Supplement: Supplementary file 3 — 10.1186/s13068-015-0370-4 Sugar consumption by S. cerevisiae null mutants expressing Y. lipolytica hexose transporters after 72 h of growth in YNB medium containing 1 % glucose (■) or 1 % galactose (■) (A). Expression profiles of Y. lipolytica genes encoding hexose transporters in the W29 and Y4588 strains (B). Yeast were incubated for 3 h in YNB medium containing 1.0 % glucose or 1.0 % galactose. The amplification of the PCR fragment in the genomic DNA served as a control for the primers’ efficiency. Abbreviations: In - inoculum. [file 13068_2015_370_MOESM3_ESM.docx]

**B**

**A**

**Additional file 3.** Sugar consumption by *S. cerevisiae* null mutants expressing *Y. lipolytica* hexose transporters after 72 h of growth in YNB medium containing 1% glucose (■) or 1% galactose (■) (A). Expression profiles of *Y. lipolytica* genes encoding hexose transporters in the W29 and Y4588 strains (B). Yeast were incubated for 3 hours in YNB medium containing 1.0% glucose or 1.0% galactose. The amplification of the PCR fragment in the genomic DNA served as a control for the primers’ efficiency. Abbreviations: In - inoculum.
