# Supplementary material for: Awakening the endogenous Leloir pathway for efficient galactose utilization by Yarrowia lipolytica
Source: Biotechnol Biofuels. 2015 Nov 25;8:185. doi: 10.1186/s13068-015-0370-4 (PMC4659199; doi:10.1186/s13068-015-0370-4)
Supplement: Supplementary file 4 — 10.1186/s13068-015-0370-4 Bacteria and yeast strains used in this study. [file 13068_2015_370_MOESM4_ESM.docx]

**Additional file 5.** Bacteria and yeast strains used in this study.

| **Name** | **Relevant genotype** | **Reference** |
| --- | --- | --- |
| ***E. coli* strains** | | |
| **JME547** | pUB4-CRE 1 | [35] |
| **JME2110** | JMP62pTEF-scGAL1-URA3ex | This study |
| **JME2111** | JMP62pTEF-scGAL1-LEU2ex | This study |
| **JME2114** | JMP62pTEF-scGAL7-URA3ex | This study |
| **JME2115** | JMP62pTEF-scGAL7-LEU2ex | This study |
| **JME2116** | JMP62pTEF-scGAL10-URA3ex | This study |
| **JME2117** | JMP62pTEF-scGAL10-LEU2ex | This study |
| **JME2542** | JMP62pTEF-ylGAL1-URA3ex | This study |
| **JME2543** | JMP62pTEF-ylGAL7-URA3ex | This study |
| **JME2544** | JMP62pTEF-ylGAL10E-URA3ex | This study |
| **JME2545** | JMP62pTEF-ylGAL10M-URA3ex | This study |
| **JME2546** | JMP62pTEF-ylGAL1-LEU2ex | This study |
| **JME2547** | JMP62pTEF-ylGAL7-LEU2ex | This study |
| **JME2548** | JMP62pTEF-ylGAL10E-LEU2ex | This study |
| **JME2549** | JMP62pTEF-ylGAL10M-LEU2ex | This study |
| **JME2735** | pRS426URA3-ylGAL1 | This study |
| **JME2736** | pRS426URA3-ylGAL7 | This study |
| **JME2738** | pRS426URA3-ylGAL10E | This study |
| **JME2739** | pRS426URA3-ylGAL10M | This study |
| **ZLE19** | pKS ylGAL10E-PUT cassette | This study |
| ***Y. lipolytica* strains** | | |
| **W29** | *MAT*a WT | [27] |
| **PO1d** | W29 *ura3-302 leu2-270 xpr2-322+pXPR2-SUC2* | [27] |
| **Y3671** | *MATa ura3-302 leu2-270 xpr2-322 pTEF-scGAL1-LEU2ex* | This study |
| **Y3681** | *MATa ura3-302 leu2-270 xpr2-322 pTEF-scGAL7-URA3ex* | This study |
| **Y3682** | *MATa ura3-302 leu2-270 xpr2-322 pTEF-scGAL10-URA3ex* | This study |
| **Y3683** | *MATa ura3-302 leu2-270 xpr2-322 pTEF-scGAL1-LEU2ex pTEF-scGAL7-URA3ex* | This study |
| **Y3684** | *MATa ura3-302 leu2-270 xpr2-322 pTEF-scGAL1-LEU2ex pTEF-scGAL10-URA3ex* | This study |
| **Y3685** | *MATa ura3-302 leu2-270 xpr2-322 pTEF-scGAL7-URA3ex pTEF-scGAL10-LEU2ex* | This study |
| **Y3686** | *MATa ura3-302 leu2-270 xpr2-322 pTEF-scGAL1 pTEF-scGAL7* | This study |
| **Y3687** | *MATa ura3-302 leu2-270 xpr2-322 pTEF-scGAL1 pTEF-scGAL7 pTEF-scGAL10-URA3ex* | This study |
| **Y4571** | *MATa ura3-302 leu2-270 xpr2-322 pTEF-scGAL1 pTEF-scGAL7 pTEF-scGAL10-URA3ex* *LEU2* clone1 | This study |
| **Y4572** | *MATa ura3-302 leu2-270 xpr2-322 pTEF-scGAL1 pTEF-scGAL7 pTEF-scGAL10-URA3ex* *LEU2* clone5 | This study |
| **Y4573** | *MATa ura3-302 leu2-270 xpr2-322 pTEF-ylGAL1-URA3ex* | This study |
| **Y4574** | *MATa ura3-302 leu2-270 xpr2-322 pTEF-ylGAL7-URA3ex* | This study |
| **Y4575** | *MATa ura3-302 leu2-270 xpr2-322 pTEF-ylGAL10E-URA3ex* | This study |
| **Y4576** | *MATa ura3-302 leu2-270 xpr2-322 pTEF-ylGAL10M-URA3ex* | This study |
| **Y4577** | *MATa ura3-302 leu2-270 xpr2-322 pTEF-ylGAL1-URA3ex pTEF-ylGAL7-LEU2ex* | This study |
| **Y4578** | *MATa ura3-302 leu2-270 xpr2-322 pTEF-ylGAL1-URA3ex pTEF-ylGAL10E-LEU2ex* | This study |
| **Y4579** | *MATa ura3-302 leu2-270 xpr2-322 pTEF-ylGAL1-URA3ex pTEF-ylGAL10M-LEU2ex* | This study |
| **Y4580** | *MATa ura3-302 leu2-270 xpr2-322 pTEF-ylGAL7-URA3ex pTEF-ylGAL10E-LEU2ex* | This study |
| **Y4581** | *MATa ura3-302 leu2-270 xpr2-322 pTEF-ylGAL7-URA3ex* *pTEF-ylGAL10M-LEU2ex* | This study |
| **Y4582** | *MATa ura3-302 leu2-270 xpr2-322 pTEF-ylGAL10E-URA3ex* *pTEF-ylGAL10M-LEU2ex* | This study |
| **Y4583** | *MATa ura3-302 leu2-270 xpr2-322 pTEF-ylGAL1 pTEF-ylGAL7* | This study |
| **Y4584** | *MATa ura3-302 leu2-270 xpr2-322 pTEF-ylGAL1 pTEF-ylGAL10E* | This study |
| **Y4585** | *MATa ura3-302 leu2-270 xpr2-322 pTEF-ylGAL1 pTEF-ylGAL7* *pTEF-ylGALl10E-LEU2ex* | This study |
| **Y4586** | *MATa ura3-302 leu2-270 xpr2-322 pTEF-ylGAL1 pTEF-ylGAL7* *pTEF-ylGAL10M-LEU2ex* | This study |
| **Y4587** | *MATa ura3-302 leu2-270 xpr2-322 pTEF-ylGAL1 pTEF-ylGAL10E pTEF-ylGAL10M-LEU2ex* | This study |
| **Y4588** | *MATa ura3-302 leu2-270 xpr2-322 pTEF-ylGAL1 pTEF-ylGAL7* *pTEF-ylGALl10E-LEU2ex* *pTEF-ylGAL10M-URA3ex* | This study |
| **Y4629** | *MATa ura3-302 leu2-270 xpr2-322 pTEF-ylGAL1-URA3ex LEU2* | This study |
| **Y4630** | *MATa ura3-302 leu2-270 xpr2-322 pTEF-ylGAL7-URA3ex LEU2* | This study |
| **Y4631** | *MATa ura3-302 leu2-270 xpr2-322 pTEF-ylGAL10E-URA3ex LEU2* | This study |
| **Y4632** | *MATa ura3-302 leu2-270 xpr2-322 pTEF-ylGAL10M-URA3ex LEU2* | This study |
| **Y4633** | *MATa ura3-302 leu2-270 xpr2-322 pTEF-ylGAL1 pTEF-ylGAL7* *pTEF-ylGALl10E-LEU2ex URA3ex* | This study |
| **Y4634** | *MATa ura3-302 leu2-270 xpr2-322 pTEF-ylGAL1 pTEF-ylGAL7* *pTEF-ylGAL10M-LEU2ex URA3ex* | This study |
| **Y4635** | *MATa ura3-302 leu2-270 xpr2-322 pTEF-ylGAL1 pTEF-ylGAL10E* *pTEF-ylGAL10M-LEU2ex URA3ex* | This study |
| **YLZ68** | *W29 ura3-302 leu2-270 xpr2-322+pXPR2-SUC2 ylgal10E::URA3ex LEU2* | This study |
| ***S. cerevisiae* strains** | | |
| **Y4473** | *Mat a; his3Δ1; leu2Δ0; met15Δ0; ura3Δ0; YBR018c::kanMX4 (ΔGal7)* | Euroscarf (Y13155) |
| **Y4474** | *Mat  a; his3Δ1; leu2Δ0; lys2Δ0; ura3Δ0; YBR019c::kanMX4 (ΔGal10)* | Euroscarf (Y13156) |
| **Y4475** | *Mat  a ; his3Δ1; leu2Δ0; lys2Δ0; ura3Δ0; YBR020w::kanMX4 (ΔGal1)* | Euroscarf (Y13157) |
| **Y4846** | *Mat  a ; his3Δ1; leu2Δ0; lys2Δ0; ura3Δ0;YDR009w::kanMX4 (ΔGal3)* | Euroscarf (Y13949) |
| **Y4591** | *Mat  a ; his3Δ1; leu2Δ0; lys2Δ0; ura3Δ0; YBR020w::kanMX4 URA3-EV(pRS426TEF)* | This study |
| **Y4592** | *Mat  a ; his3Δ1; leu2Δ0; lys2Δ0; ura3Δ0; YPL248c::kanMX4 URA3-EV(pRS426TEF)* | This study |
| **Y4593** | *Mat a; his3Δ1; leu2Δ0; met15Δ0; ura3Δ0; YBR018c::kanMX4 URA3-EV(pRS426TEF)* | This study |
| **Y4594** | *Mat  a ; his3Δ1; leu2Δ0; lys2Δ0; ura3Δ0; YBR019c::kanMX4 URA3-EV(pRS426TEF)* | This study |
| **Y4595** | *Mat  a ; his3Δ1; leu2Δ0; lys2Δ0; ura3Δ0; YBR020w::kanMX4 pTEF-ylGAL1-URA3* | This study |
| **Y4596** | *Mat a; his3Δ1; leu2Δ0; met15Δ0; ura3Δ0; YBR018c::kanMX4 pTEF-ylGAL7-URA3* | This study |
| **Y4597** | *Mat  a ; his3Δ1; leu2Δ0; lys2Δ0; ura3Δ0; YBR019c::kanMX4 pTEF-ylGAL10E-URA3* | This study |
| **Y4690** | *Mat  a ; his3Δ1; leu2Δ0; lys2Δ0; ura3Δ0; YBR019c::kanMX4 pTEF-ylGAL10M-URA3* | This study |
| **Y4874** | *Mat  a ; his3Δ1; leu2Δ0; lys2Δ0; ura3Δ0;YDR009w::kanMX4 URA3-EV(pRS426TEF)* | This study |
| **Y4875** | *Mat  a ; his3Δ1; leu2Δ0; lys2Δ0; ura3Δ0;YDR009w::kanMX4 URA3-ylGAL1(ΔGal3)* | This study |
| **B01342** | *leu2-3,112 ura3-52 trp1-289 his31 MAL2-8C SUC2 hxt17 hxt13 hxt15 hxt16 hxt14 hxt12 hxt9 hxt11 hxt10 hxt8 hxt514 hxt2 hxt367 gal2 slt1 agt1 ydl247 yjr160c URA3-YALI0B01342* | Lazar et al., in preparation |
| **B06391** | *leu2-3,112 ura3-52 trp1-289 his31 MAL2-8C SUC2 hxt17 hxt13 hxt15 hxt16 hxt14 hxt12 hxt9 hxt11 hxt10 hxt8 hxt514 hxt2 hxt367 gal2 slt1 agt1 ydl247 yjr160c URA3-YALI0B06391* | Lazar et al., in preparation |
| **C06424** | *leu2-3,112 ura3-52 trp1-289 his31 MAL2-8C SUC2 hxt17 hxt13 hxt15 hxt16 hxt14 hxt12 hxt9 hxt11 hxt10 hxt8 hxt514 hxt2 hxt367 gal2 slt1 agt1 ydl247 yjr160c URA3-YALI0C06424* | Lazar et al., in preparation |
| **C08943** | *leu2-3,112 ura3-52 trp1-289 his31 MAL2-8C SUC2 hxt17 hxt13 hxt15 hxt16 hxt14 hxt12 hxt9 hxt11 hxt10 hxt8 hxt514 hxt2 hxt367 gal2 slt1 agt1 ydl247 yjr160c URA3-YALI0C08943* | Lazar et al., in preparation |
| **E23287** | *leu2-3,112 ura3-52 trp1-289 his31 MAL2-8C SUC2 hxt17 hxt13 hxt15 hxt16 hxt14 hxt12 hxt9 hxt11 hxt10 hxt8 hxt514 hxt2 hxt367 gal2 slt1 agt1 ydl247 yjr160c URA3-YALI0E23287* | Lazar et al., in preparation |
| **F19184** | *leu2-3,112 ura3-52 trp1-289 his31 MAL2-8C SUC2 hxt17 hxt13 hxt15 hxt16 hxt14 hxt12 hxt9 hxt11 hxt10 hxt8 hxt514 hxt2 hxt367 gal2 slt1 agt1 ydl247 yjr160c URA3-YALI0F19184* | Lazar et al., in preparation |
