# Supplementary material for: Awakening the endogenous Leloir pathway for efficient galactose utilization by Yarrowia lipolytica
Source: Biotechnol Biofuels. 2015 Nov 25;8:185. doi: 10.1186/s13068-015-0370-4 (PMC4659199; doi:10.1186/s13068-015-0370-4)
Supplement: Supplementary file 5 — 10.1186/s13068-015-0370-4 Construction of the Y. lipolytica strains in which the scGAL genes were overexpressed. The auxotrophic PO1d strain was used as the acceptor strain. The genes were inserted one by one to create strains that overexpressed different combination of the scGAL genes; URA3ex and LEU2ex were used as selection markers. To recover auxotrophies necessary to continuing the transformation process, a JME547 plasmid containing Cre-Lox recombinase was used to transform Y. lipolytica Y3683, thus generating strain Y3686 [35]. In turn, to recover prototrophy in the Y. lipolytica Y3687 strain, a purified SalI fragment of the pINA62 plasmid that contained the LEU2 gene was introduced [28]. [file 13068_2015_370_MOESM5_ESM.docx]

**Y195**

**Y3681**

**Y3682**

**Y3671**

**Y3683**

**Y3686**

**Y3687**

**Y3684**

**Y3685**

*scGAL1*

*LEU2ex*

*scGAL10*

*URA3ex*

*scGAL7*

*URA3ex*

*scGAL7*

*URA3ex*

*scGAL10*

*URA3ex*

*scGAL10*

*LEU2ex*

*scGAL10*

*URA3ex*

*CreLox*

**Y4571**

*LEU2*

**Additional file 4.** Construction of the *Y. lipolytica* strains in which the *scGAL* genes were overexpressed. The auxotrophic PO1d strain was used as the acceptor strain. The genes were inserted one by one to create strains that overexpressed different combination of the *scGAL* genes; URA3ex and LEU2ex were used as selection markers. To recover auxotrophies necessary to continuing the transformation process, a JME547 plasmid containing Cre-Lox recombinase was used to transform *Y. lipolytica* Y3683, thus generating strain Y3686 [35]. In turn, to recover prototrophy in the *Y. lipolytica* Y3687 strain, a purified *Sal*I fragment of the pINA62 plasmid that contained the *LEU2* gene was introduced [28].
