# Supplementary material for: Awakening the endogenous Leloir pathway for efficient galactose utilization by Yarrowia lipolytica
Source: Biotechnol Biofuels. 2015 Nov 25;8:185. doi: 10.1186/s13068-015-0370-4 (PMC4659199; doi:10.1186/s13068-015-0370-4)
Supplement: Supplementary file 6 — 10.1186/s13068-015-0370-4 List of primers used in this study. [file 13068_2015_370_MOESM6_ESM.docx]

**Additional file 6.** List of primers used in this study.

| No | Primer | Sequence | Restriction site | Amplified gene |
| --- | --- | --- | --- | --- |
| OE29 | Gal1-BamHI-fwd | gcgc**ggatcc**atgactaaatctcattcagaagaagtgattg | BamHI | *scGAL1* |
| OE30 | Gal1-AvrII-rev | gcgc**cctagg**ttataattcatatagacagctgcccaatgc | AvrII |  |
| OE35 | ScGal7-BamHI-fwd | gcgc**ggatcc**atgactgctgaagaatttg | BamHI | *scGAL7* |
| OE36 | Gal7-AvrII-rev | gcgc**cctagg**ttacagtctttgtagataatgaatc | AvrII |  |
| OE37 | Gal10-BamHI-fwd | gcgc**ggatcc**atgacagctcagttacaaag | BamHI | *scGAL10* |
| OE38 | Gal10-AvrII-rev | gcgc**cctagg**tcaggaaaatctgtagacaatc | AvrII |  |
| OE98 | YLGal1Short-BamHI-fwd | gtga**ggatcc**atgtctatcagcaccctgcc | BamHI | *ylGAL1* |
| OE40 | YlGal1-AvrII-rev | gcgc**cctagg**ttacaaatcaacaatagcacatccc | AvrII |  |
| OE41 | YlGal7-BamHI-fwd | gaga**ggatcc**atgactcttgtggcatccg | BamHI | *ylGAL7* |
| OE42 | YlGal7-AvrII-rev | gcgc**cctagg**ttacaaatcatacaaccgcttgg | AvrII |  |
| OE43 | YlGal10E-BglII-fwd | gaga**agatct**atgacccaggctgctgctgaac | BglII | *ylGAL10E* |
| OE44 | YlGal10E-SpeI-rev | gcgc**actagt**ttacttgtcatgacggtcctttttgtaacc | SpeI |  |
| OE45 | YlGal10M-BamHI-fwd | gaga**ggatcc**atgaccttttgcaacacgggagcaac | BamHI | *ylGAL10M* |
| OE46 | YlGal10M-AvrII-rev | gcgc**cctagg**tcactcccgctccaaaacatagataatcttagc | AvrII |  |
| OE99 | YLGal1Short-SpeI-fwd | gcgc**actagt**atgtctatcagcaccctgcc | SpeI | *ylGAL1* |
| OE89 | YlGal1-SmaI-rev | gcgc**cccggg**ttacaaatcaacaatagcacatccctc | SmaI |  |
| OE90 | YlGal7-SpeI-fwd | gaga**actagt**atgactcttgtggcatccg | SpeI | *ylGAL7* |
| OE91 | YlGal7-SmaI-rev | gcga**cccggg**ttacaaatcatacaaccgcttggc | SmaI |  |
| OE92 | YlGal10E-SpeI-fwd | gaga**actagt**atgacccaggctgctgctg | SpeI | *ylGAL10E* |
| OE93 | YlGal10E-SmaI-rev | gaga**cccggg**ttacttgtcatgacggtcctttttg | SmaI |  |
| OE100 | YLGal10M-SpeI-fwd | gcgc**actagt**atgaccttttgcaacacgggagcaac | SpeI | *ylGAL10M* |
| OE101 | YLGal10M-EcoRI-rev | gcgc**gaattc**tcactcccgctccaaaacatagataatcttag | EcoRI |  |
| ylGAL10E-P1 | | gaga**gcggccgc**gaagcacatatcaataaggagaac | NotI | *ylGAL10E* |
| ylGAl10E-P2 | | cg**attaccctgttatcccta**cccgtgctgcggtgttagttg | I-SceI |  |
| ylGAl10E-T1 | | gg**tagggataacagggtaat**cgaggaccgtcatgacaagtaag | I-SceI |  |
| ylGA10E-T2 | | gaga**gcggccgc**tgcatcggagatggattttg | NotI |  |
| Primers used for RT-PCR and qRT-PCR | | | | |
| YlGal1-RT-F | | tcttcaactgctcctgtcct | *ylGAL1* | |
| YlGal1-RT-R | | ctatcgtaatcgacaccatcc |  |  |
| YlGal7-RT-F | | ccatcaaaaagctgaccaag | *ylGAL7* | |
| YlGal7-RT-R | | atcgcagaagaggagggtag |  |  |
| YlGal10E-RT-F | | tggtgacgactacccctct | *ylGAL10E* | |
| YlGal10E-RT-R | | cggttcctaggttccattct |  |  |
| YlGal10M-RT-F | | tccatctccctcaatggaac | *ylGAL10M* | |
| YlGal10M-RT-R | | gccggtatagacctggaaac |  |  |

*The primers used to analyze hexose transporter expression will be included in a future publication (Lazar et al., in preparation).
